# Keeping in Touch with Mental Health: The Orienting Reflex and Behavioral Outcomes from Calatonia

**DOI:** 10.3390/brainsci10030182

**Published:** 2020-03-22

**Authors:** Anita Ribeiro Blanchard, William Edgar Comfort

**Affiliations:** 1 Faculty of Psychology, University of Barcelona, 08035 Barcelona, Spain; 2 Social and Cognitive Science Laboratory, Centre for Health and Biological Sciences, Mackenzie Presbyterian University, São Paulo 01241, Brazil; 9032936@mackenzie.br

**Keywords:** orienting reflex, motivational system, touch therapy, integrative psychotherapy, somatic psychology

## Abstract

Physical and psychological therapy based on touch has been gradually integrated into broader mental health settings in the past two decades, evolving from a variety of psychodynamic, neurobiological and trauma-based approaches, as well as Eastern and spiritual philosophies and other integrative and converging systems. Nevertheless, with the exception of a limited number of well-known massage therapy techniques, only a few structured protocols of touch therapy have been standardized and researched to date. This article describes a well-defined protocol of touch therapy in the context of psychotherapy—the Calatonia technique—which engages the orienting reflex. The orienting reflex hypothesis is explored here as one of the elements of this technique that helps to decrease states of hypervigilance and chronic startle reactivity (startle and defensive reflexes) and restore positive motivational and appetitive states.

## 1. Introduction

The limitations of verbal psychotherapy have become more evident in the past thirty years [1], giving rise to a large number of somatic and body-based modalities aimed to address treatment-resistant disorders [2,3,4]. Recently, there has been an interest in developing somatically informed research methods to support a wide range of these integrative practices [5,6]. Accordingly, this article discusses the significance of integrating a structured touch therapy (Calatonia) into psychotherapy to facilitate an orienting reflex (OR) [7]. The OR leads the individual to direct their motivational system towards appetitive and exploratory states, which can, in turn, positively influence affective and cognitive states.

Motivation—a concept derived from the biological sciences—has not been explored for its potential strength in mental health treatments, although motivation as a cognitive concept was well developed by Miller [8]. Affective responses comprising an organism’s underlying motivational state have been broadly categorized in terms of defensive and appetitive systems, evolving either separately or in conjunction to engage with environmental stimuli indicative of threat or opportunities for survival, respectively [9]. In this perspective, emotional experiences occur within a range of appetitive-pleasant or defensive-unpleasant valence and have levels of arousal that indicate the degree of activation in response to that emotional valence [10]. These two basic dimensions of affective responses support mobilization for action, attention and social communication, according to the motivational system that is engaged (defensive or appetitive), its intensity of activation and its emotional context [9,10,11,12,13].

While several findings have identified distinct brain networks for approach/avoidance behavior and pleasant or aversive affect in healthy subjects [14,15], mental health continues to be studied primarily from a symptomatological perspective, with little research into long-term behavioral outcomes linked to the patient’s motivational state.

As an illustration, depressive disorders—from their biological symptoms to their emotional and cognitive expression—can be understood as dysfunctions of the motivational system, in which (appetitive) motivation is reduced. Anxiety disorders may also be viewed from a motivational system perspective, in which there is either strong behavioral inhibition or impulsivity, based on aversive, defensive or avoidant motivation [16]. In such cases, touch therapies may prove useful to redirect the individual’s motivational state toward more approach-oriented behavior, in conjunction with conventional psychotherapy and/or pharmacological treatment.

It is in this context that we introduce the therapeutic potential of Calatonia, a long-standing technique of touch therapy which aims to re-orient the individual toward a more open behavioral approach through activation of the appetitive motivational system and concomitant inhibition of startle and defensive states. This integrative approach has been used to treat disorders unresponsive to verbal psychotherapy alone, such as PTSD and other forms of trauma.

The primary mechanism through which Calatonia is thought to exert changes in the individual’s motivational states is by eliciting an orienting reflex (OR) within the context of psychotherapy [17]. The OR is activated through touch experienced as a novel, sustained and non-threatening stimulus. Calatonia (described in Section 3) is a therapeutic technique based on a structured sequence of touches applied bilaterally to distinct regions of the body [18,19,20]. Since its inception, Calatonia has purported to induce a state of deep relaxation and increased unconscious processing with a net result of altering the patient’s motivational and affective states [19,20,21].

However, Calatonia has yet to be submitted to rigorous scientific testing for the direct therapeutic benefits accrued from its application. One potential avenue for such research is the Research Domain Criteria (RDoC) framework for evaluating novel treatments for mental health put forth by the National Institute of Mental Health (NIMH) [22]. Within the RDoC framework, an upward level of analysis constitutes mapping functional measures of neural activity to variation in clinical symptoms on a distinct spectrum of mental health such as anxiety [22]. In line with this approach, a study utilizing near-infrared spectroscopy (fNIRS) to investigate alterations in neural markers of anxiety following Calatonia is currently under preparation.

## 2. History of the Technique

During WWII, the Hungarian physician Pethö Sándor (1916–1992) structured a sequence of ten light touches while treating the psychological and physical suffering of refugees and other displaced persons at Red Cross refugee camps. This sequence of touches emerged from the combined biomedical knowledge and feedback from patients about the points of tactile contact that appeared to balance their sympathetic and parasympathetic responses and foster autonomic regulation [18,19,20].

After being treated with this technique—then named Calatonia—patients spontaneously shared their feelings, thoughts, worries, memories and traumatic experiences with a scale of trust and openness that had not happened before the treatment—a clear validation of its usefulness in psychotherapy [19,20]. Following a few applications of Calatonia, patients showed decreased symptoms of traumatic stress (shell shock), anxiety, depression, pain and other ailments that afflicted most war survivors at the time. Patients’ improvements manifested in terms of increased morale, acceptance, well-being, hope, will to live, overall motivation and resilience [21], defined as the ability to adequately adapt and respond to homeostatic disturbances [23]. These touches seemed to promote global changes, which led Sándor [19,20] to describe Calatonia as a technique for psychophysical regulation and reorganization.

After the end of the war, Sándor worked for two years in the psychiatric wards of German hospitals, using Calatonia to successfully treat depression, suicide ideation, post-traumatic stress disorder, anxiety, catatonic states and other mental disorders [18,21,24,25]. Later, in São Paulo, Brazil [26], he expanded the repertoire of techniques to include many other “light touch” sequences, grouped under the name subtle touch (ST) [27,28,29,30]. Another ST technique, Fractional Decompression, works by gradually releasing pressure from a touch applied to hairy skin on the back, arms or legs [27]. Fractional decompression is thought to primarily target the affective-affiliative touch system associated with hairy non-glabrous skin [31]. Calatonia continues to be the most widely used ST technique. At times, subtle touch and Calatonia are used interchangeably to denote the whole gamut of techniques developed by Sándor and subsequently expanded by other clinical psychologists [32]. Sándor’s subtle touch method has produced numerous qualitative studies published over the past four decades (reviewed in [32]), as well as quantitative research in the past fifteen years [32,33,34].

Sándor had previously hypothesized [19,20] that the experience of physiological regulation, mood stabilization, inflow of adaptive cognition and neuromuscular relaxation induced by Calatonia were linked to the associative activation of somatosensory representations in the frontotemporal cortex, the engagement of peripheral proprioceptive nerve fibers, particularly in the skin and cortical mediation by the ascending reticular activating system. Furthermore, he associated its effects with psycho-affective elements mobilized by the configuration of dyadic regulation through the touch therapy protocol.

Given the barriers to many forms of social and affiliative touch in social interactions, particularly in the context of psychotherapy, it can be useful to compare the touch sequence employed in Calatonia with other common forms of “pleasant” touch found in everyday encounters. In particular, a recently discovered category of slow-response unmyelinated nerve fiber, C-tactile afferents, have been implicated in many forms of innocuous touch [35], as well as touch in social contexts [31]. C-tactile afferent projections terminate in the ventral medial nucleus of the thalamus and posterior insular cortex [36,37], associated with the contextual and affective components of touch [38]. While the primary areas of contact in Calatonia are to the glabrous skin areas of the feet or hands (see below), the sequence employed also includes contact to hairy skin containing C-tactile afferent connections. Several other ST techniques similarly activate CT connections by contact with the arms, calves, back and head. As such, Calatonia may act on both a common affiliative system for social touch as well as on more discriminative neural pathways in glabrous areas of the hands and feet. However, the perceptual characteristics of touch in these regions are specifically modulated in Calatonia to induce large-scale novelty-related activation in addition to more familiar responses to CT touch primarily in thalamic and insular regions.

## 3. The Calatonia Technique

Touch therapies differ in their goals. Some focus on achieving body awareness, structural readjustment, functional improvement, emotional-affective regulation, release of pent up energy, healing of trauma, among other issues [2]. Calatonia has an open-ended goal, in contrast to more narrowly defined ST techniques: one geared toward spontaneous adaptive adjustments in one’s idiosyncratic psychophysical needs and imbalances, prompted by the sequence of touches. As an example, for some, stress will manifest as insomnia or excessive worrying; for others, it will manifest as addiction, overeating or overreacting emotionally in relationships. Each maladaptive style will lead to different responses to Calatonia’s applications, despite being caused by the same underlying problem—stress.

**Figure 1:**
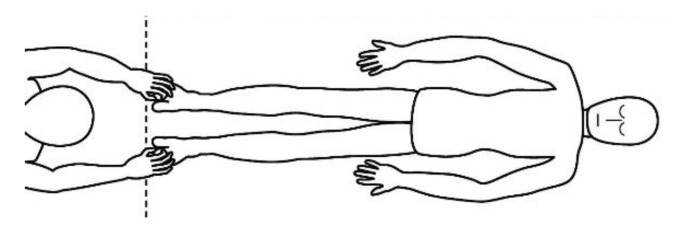
Resting state (task-free) supine position.

**Figure 2:**
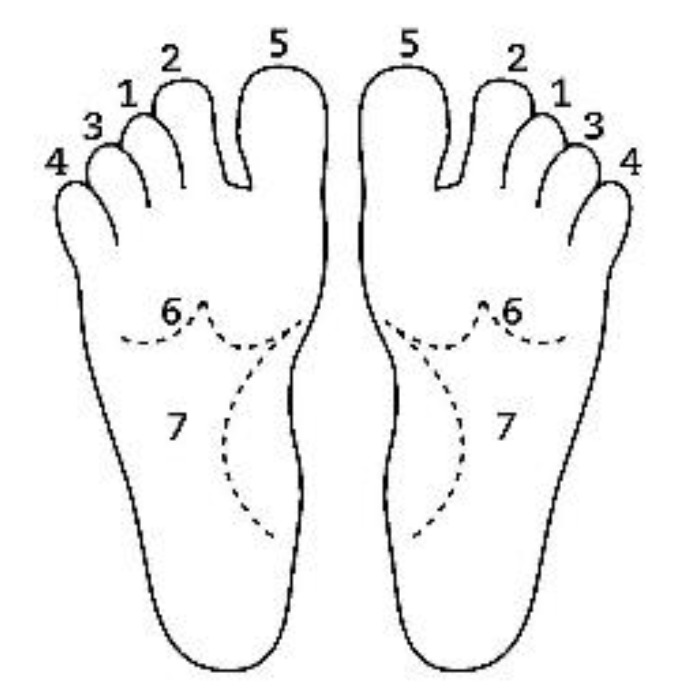
Sequential order of bilateral points of tactile contact.

**Figure 3:**
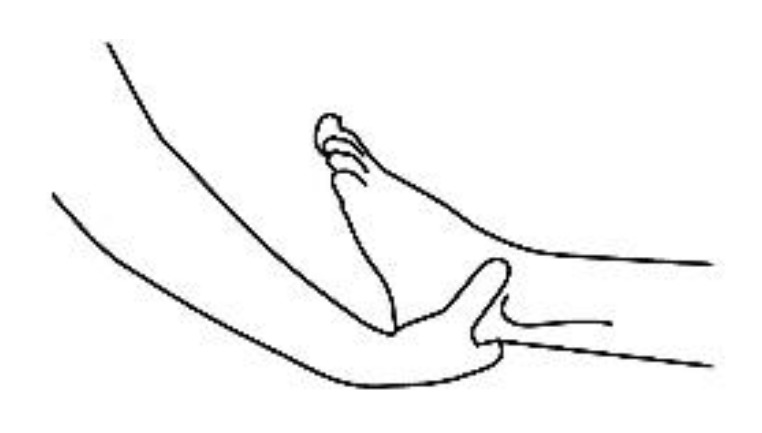
Ankle support, eighth bilateral touch.

**Figure 4:**
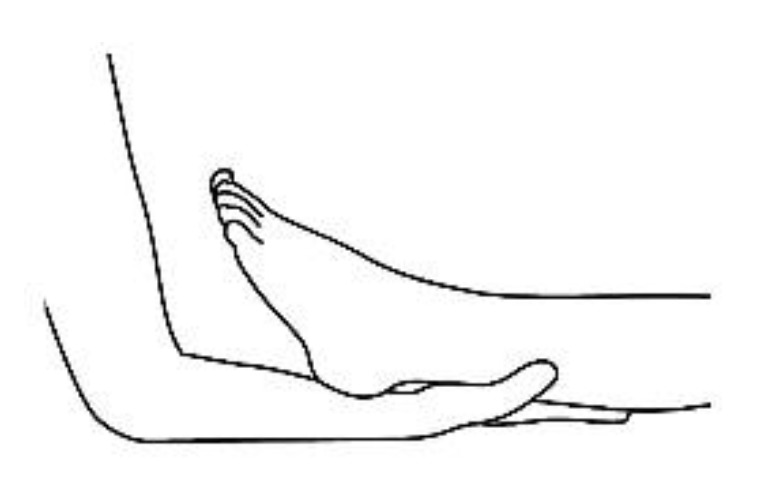
Calf support, ninth bilateral touch.

**Figure 5:**
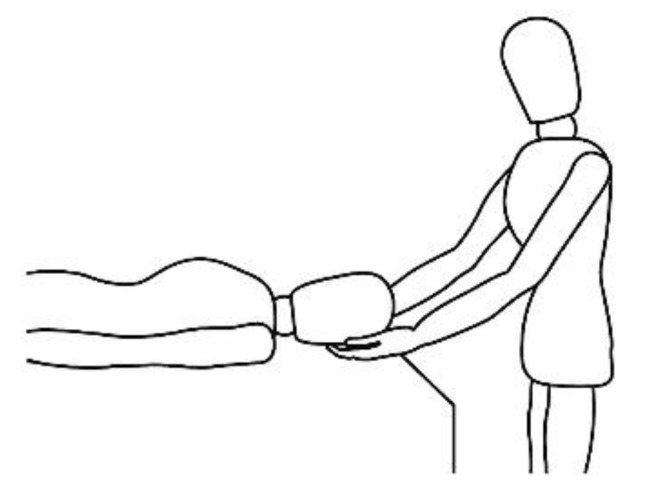
Head support, tenth and last touch.

A description of this technique (Figure 1, Figure 2, Figure 3, Figure 4 and Figure 5) may be useful to integrate the elements that will be discussed in this article. Calatonia is performed in silence (unless the patient feels discomfort or the need to speak) after the patient has been briefed on the steps of the protocol [19,20]. The patient removes his or her shoes and socks and lies on a massage table in a supine position, with his or her eyes closed, fully dressed (Figure 1). Preferably, the therapist applies the technique on the lower limbs, or, alternatively, on the hands and forearms. Excluding the tenth point (the head), the first nine points of tactile contact are bilateral (the same tactile stimulus is simultaneously held on each side of the body). The seven first touches (Figure 2) are extremely light (as if the therapist were holding a “soap bubble”) and sustained in place for one to three minutes (preferably three minutes on the few initial applications). The eighth, ninth and tenth tactile contacts (respectively Figure 3, Figure 4 and Figure 5) are supported and held in place for one to three minutes on the heels, calves and head (in that order). At the end, patients are coached back to awareness of the environment and themselves and instructed to sit up and walk back to their seat, at which point they are invited to share their observations or impressions, if any, which may have manifested during or after the application of the technique.

## 4. Touch to the Feet or Hands

While it appears unusual to propose tactile contact with the patient’s feet or hands in psychotherapy, there is strong empirical support for doing so. Contact is made to these specific sites, as nowhere else on the body is there found a similar configuration of neurobiological and physiological variables, including the distinctive dermo-mechanical features and receptors found on the glabrous skin of the feet and hands [35,36,37,38,39,40,41,42,43,44]. This combination of features frequently results in the activation of an orienting response by eliciting a pattern of neural activation associated with novelty, of either a neutral or pleasant nature, through both conscious and unconscious perceptual pathways.

The glabrous (non-hairy) skin of the hands and feet is indirectly connected to several perceptual subsystems involved in the detection of vibration, temperature changes and differences in texture and pressure, as well as somatosensory and proprioceptive responses [43,44]. The areas targeted in Calatonia contain the most numerous populations of skin receptors, collectively known as the discriminative–spatial system [41,42,43,44,45,46], distinct from receptors of the affective–affiliative system found primarily in non-glabrous skin. Glabrous skin is a dedicated site of very precise tactile perception geared toward the exploration of and adaptation to novel stimuli, as well as the evaluation and appraisal of a spectrum of touch pleasantness and roughness mediated by the somatosensory cortex [47,48,49].

For example, to read and attribute meaning to the raised dots of the Braille system requires language, touch and spatial coding to be transformed into semantic, lexical and haptic processing. This in turn engages highly associative areas of the brain to produce concrete and abstract thinking, symbols and ultimately communication [50].

Hands are also especially involved in the formation of procedural memory, which makes them potential “roads” for the emotional retrieval of such implicit memories—in particular, early childhood memories linked to independence, mastery, self-care, reaching out and so forth. These memories may be accompanied by emotional and relational contexts of frustration, impatience, among many other emotions and behavioral patterns [51,52]. Similarly, the locomotor system is in many ways involved in early childhood developmental milestones (standing, walking, running, bike riding, etc.).

The feet bear the total gravitational force imposed on the body and function as an integrated system with the cerebellum and vestibular system to control posture, coordination, equilibrium and the generation of locomotor rhythm; the proprioceptive control of posture is chiefly initiated in the feet [53]. Drew, Prentice and Schepens [54] state that these essential mechanisms of control of postural muscle tone and locomotion “are located in the brainstem and spinal cord, in which a range of locomotor behaviors are achieved by the projections from the forebrain structures (cerebral cortex, basal ganglia and limbic-hypothalamic systems) and cerebellum to the brainstem-spinal cord”. As such, despite the role of these mechanisms in voluntary movement and locomotion, a significant portion of the adjustment of balance is made involuntarily, based on information that does not require conscious attention to be processed. When the feet are in an unloaded position (i.e., lying down), there is no background discharge activity in any of the cutaneous receptors unless there is intentionally applied stimulation [49,53,54,55]. This may be indicative of how Calatonia on the feet facilitates the reorganization of the individual’s global posture and muscle tension.

## 5. Novel Stimuli in Psychotherapy

There are several common elements to Sándor’s many ST techniques, yet two elements can be immediately perceived as fundamental: (a) their non-invasiveness, by respecting an individual’s boundaries and even their resistance to therapy, while gently supporting the individual towards gaining resilience toward the integration of crucial personal issues; (b) their novelty, through the application of atypical sensory stimuli—not merely an oddball protocol for the sake of novelty itself, but a meaningful stimulus that engages global responses and multidimensional aspects of a person’s life.

The combination of these two aspects, non-invasiveness and novelty, is hypothesized as generating an orienting reflex or an orienting response [7], also known as the ‘what is it response?’ or the exploratory response. The OR is an involuntary response of an organism to a stimulus that is ‘out of the ordinary’ but not alarming or menacing. ORs are an adaptive feature of cognition present since infancy [56] that play a major role in many aspects of motivation, emotion and attention [57,58,59,60,61,62].

Sándor purposefully developed different methods for creating ORs by using an unusually light and static form of touch or other forms of stimulation in his techniques. These included passive movements that were mechanically impossible for the patient to perform voluntarily (e.g., rotating the patient’s finger sideways); atypical but non-threatening sounds directed towards specific parts of the body, small vibrations applied to bone projections and protuberances, such as the spinous processes [27] and many others. His approach was geared toward the enhancement of neural plasticity and the generation of ‘dedicated neural circuitries’ for experiencing well-being, leading to increased self-confidence and a sense of safety within one’s own body [17,18].

## 6. The Orienting Reflex in Calatonia

An orienting reflex is triggered when a sensory stimulus is perceived as novel, innocuous or pleasant [58,63]. This may be seen in opposition to defensive reflexes initiated when a sensory stimulus is perceived as painful, aversive and potentially dangerous—or startle reflexes activated in response to abrupt, unexpected or overly intense stimuli. All of these responses, whether defensive/startle or orienting, will activate the executive network for regulation or action if necessary [64,65].

In animals, the OR is a survival reflex that does not burden the organism with a full-blown alert response but entices them to explore the environment. Pavlov [66] saw the OR as the biological basis for the highest form of curiosity, imagination, science and knowledge of the world around us. At the basic end of this spectrum, the OR encourages human curiosity, which drives child development and “involves an indissoluble mixture of cognition and motivation” [67]—a key prerequisite for learning and the formation of top-down predictions in perceptual processing. To achieve this level of exploration, the OR tunes the organism toward a specific stimulus by enhancing perceptual awareness [68,69,70]. In contrast, the defensive and startle reflexes limit the impact of the stimulus on the organism by functionally raising perceptual thresholds [68,69,70].

The OR halts all non-essential brain activity to allow the individual to orientate their primary senses towards the source of stimulation, focusing on possible means of interaction with the stimulus through the activation of the autonomic nervous system (ANS) [68,69]. It produces an unintentional shift of attention that interrupts the ordinary flow of awareness and leads us to attend to the novelty of a stimulus for the appraisal of its meaning and/or significance. This phase of the reflex has been classified by researchers as an “information-gathering”, “analyzing”, “modelling” or simply “sensory” phase [7]. One of the key physiological markers of an OR is the initial deceleration in the heartbeat, which is a sign of enhanced perceptual processing and is mediated by the parasympathetic branch of the ANS [71,72]. This initial slowdown allows the organism to more easily detect the potential significance of stimulus features to estimate ‘uncertainty’. An OR is triggered if uncertainty is detected concerning the biological value or perceptual features of the stimulus [73].

As mentioned above, Calatonia triggers an OR due to its non-invasive nature (experienced as either of a neutral or pleasant affect), extended duration of passive tactile stimuli and the novelty of its touch. Sustained attention to a body location results in the enhanced processing of the tactile stimuli presented at that location compared to other unattended locations [74]. A light touch is often a strange sensory perception, particularly on the feet, accustomed to supporting the individual’s body weight and rough contact with stimuli on the ground. The palms of the hands and the soles of the feet are instruments of self-agency [75,76,77] and rarely the object of passive interaction. Receiving a passive gentle touch in these areas may easily throw the organism into a state of perceptive, emotional and cognitive surprise yet still feel innocuous within a safe context. The neutral affect associated with the surprise response works to reset attentional processes [78]—with attention defined in cognitive terms as the ability to selectively attend to some stimuli while ignoring others [79].

Individuals have different forms of appraisal and emotional responses to touch based on their personal history and cultural biases [80]. However, it is highly likely that most people will experience an OR in response to an unexpectedly static, sustained and light touch that conveys neither personal meaning nor affection. Such a response is dependent on the therapist adopting an appropriately responsive but neutral affective state during the application of Calatonia.

## 7. Brain Areas Associated with the Orienting Reflex

Sándor’s hypothesis concerning reticular formation mediation in the large-scale neural response activated during Calatonia has been borne out by subsequent research based on brain imaging (fMRI-PET, EEG, NIRS). Here, we articulate an updated argument in the light of this evidence for the therapeutic benefit of engaging the orienting reflex through novel stimulation and the cognitive re-evaluation this may engender.

The functional circuitry of the reticular formation (RF), known as the reticular activating system (RAS), has long been recognized as a central component within a multitude of subcortical and cortical neural circuitry [81]. The RAS has been implicated in cognitive functions such as the orienting reflex to novel stimuli [7], attention, sleep, homeostatic regulation, as well as the transmission and modulation of pain, alongside other brain structures [82,83,84,85]. Essentially, the RF plays a major role in the modulation of attention to the extremely light sensory stimuli of Calatonia, which takes the organism by surprise and induces an orienting reflex, with extensive engagement of the RF and related networks in this process.

The OR triggers an extensive search for possible associations to previous contexts and meanings within the individual’s history, beginning with short-term memories and moving on to those that may be embedded in implicit memory. With the aim of associating the new stimulus to previous memory representations, the brain quickly ‘explores’ the memory via the hippocampus and sensory association areas in the central-parietal cortex. In this process, a set of neocortical limbic interactions occur to resolve the significance of the stimulus [73,86,87].

Uddin and colleagues [88] note that the insula is commonly involved in detection of novel stimuli across sensory modalities. The insula, dorsal anterior cingulate (dACC) and other subcortical structures form part of the ‘salience network’ (SN), which is activated in response to out-of-the-ordinary or “oddball” stimuli. The function of the SN is to identify “the most homeostatically relevant among multiple competing internal and external stimuli” [88]. Most importantly, for the higher-order cognitive processes produced with Calatonia [17] where more complex stimuli require greater cognitive processing, the dorsal anterior insula will be recruited. Insular activation primarily functions “to integrate external sensory information with internal emotional and bodily state signals to coordinate brain network dynamics and to initiate switches between the default mode network (DMN) and central executive network (CEN)” [88].

If no associations are formed between the tactile stimuli in Calatonia and previous experiences recorded as memories, then the significance of the event will be assessed primarily by the amygdala. The amygdala plays an important role in encoding, storing and retrieving emotionally charged events and controlling the hormonal cascade triggered by defensive responses [89]. Amygdalar structures are activated by both emotionally salient and novel stimuli. This activation may occur regardless of whether the stimuli are emotionally valent and/or pleasant or unpleasant. In addition, the salience of the emotion is central to determining whether either a fight-or-flight or other motivational or appetitive response is triggered [90,91,92,93,94].

## 8. The Appraisal of New Stimuli

A stimulus or event is significant if it is helpful in satisfying a need, reaching a goal, or sustaining an internal value. The opposite holds true for negative significance, whereupon the stimulus is unhelpful for achieving any of the aforementioned goals. In ANS terminology, significance can be equated to homeostasis, whereby a stimulus or event positively influences homeostatic mechanisms, leading to that stimulus being attributed a high level of significance [92,95,96].

Scherer [97] asserts that the appraisal of significance is defined by one’s needs, goals, values and general wellbeing, which leads to a cascade of motivation-related changes. In particular, emotionally-laden appraisals of pleasantness and well-being (or the opposite valence) lead to somatovisceral changes via the ANS and changes in motor facial expression, as well as voice and bodily tensions, conveyed through the somatic nervous system [97,98,99]. Over the course of Calatonia, adjustments in body tension are frequently reported in the form of twitching, sudden jerks, spontaneous jolts felt in the diaphragm, lung expansion (a respiratory reflex) and fluttering eyelids, while at the end of Calatonia, the facial muscles are often notably relaxed [17,18,19,20,32].

In the presence of a pleasant OR, several somatic responses may occur, such as a deceleration in heart rate, salivation, pupillary responses, pharyngeal expansion and a relaxation of the tract walls (‘wide voice’) [97]. These many somatic reactions are conducive to a trophotropic response (a relaxation response for resting and replenishing energy) and increased stability. This may in turn lead to a decrease in respiration rate, a slight decrease in heart rate, sphincter relaxation, a decrease in general muscle tone, relaxation in facial muscle tone and overall relaxation of the vocal apparatus (‘relaxed voice’), comfort and resting posture. If this relaxation response leads to changes in one’s motivational state and plans for action, an ergotropic shift (the activation response and usage of energy) may occur as a result of experiencing well-being [97]—in this way, one feels motivated to become proactive. This fact may explain why Calatonia is a method of psychophysiological regulation and not exclusively a relaxation technique because, ultimately, it takes the organism where it needs to go. Whether positively activated (ergotropic) or relaxed (trophotropic), Calatonia fosters the organism’s optimal state [17,18,19,20].

## 9. ORs in Clinical Practice

The emotional significance of a stimulus, defined by its level of pleasantness and importance, can frequently affect OR intensity when focusing one’s attention on a stimulus [7,100,101]. One example of the use of OR in clinical practice is a simple technique designed by Sándor: a sequence of three slow and sustained ‘insufflation (blowing) on and above the seventh cervical vertebra (C4–C7)’. The therapist applies the technique while the patient remains in a sitting or standing position. This somatosensory contact at C4–C7 affects the entirety of the brachial plexus, which innervates the arm muscles, thus affecting a large area of the brain, as well as cervical vagus branching [102], causing an immediate and involuntary shift in attention with a pleasant affective tone. This is an effective way of peacefully redirecting disruptive behavior in children in less than a couple of minutes and one that has been applied on many previous occasions to institutionalized children in foster care when they felt themselves unable to engage in emotional regulation [17]. For these children, the novelty of tactile stimulation diverted their attention from an overwhelming state of emotional reaction, offering them a state of adaptive relief directly proportional to this initial state.

The process of neocortical-limbic connectivity and integration linked to an OR is not a standard occurrence in children and adults with histories of abuse and/or PTSD [103,104,105]. These patients frequently show symptoms of excessive limbic system activity (particularly an abnormally overactive amygdala) with less activity in the neocortex, which causes them to react impulsively to the minor triggers of daily life [106]. However, in several cases of PTSD, the opposite pattern of activation is observed, with over-activation of the prefrontal systems and over-inhibition of the amygdala and insula, leading these patients to experience flat affective states and anhedonia.

When the amygdala is chronically activated in response to trauma, stress and/or overwhelming fear, the individual’s emotional response to sensory inputs becomes compromised and often requires re-setting [68,69,83]. The effects of exposure to traumatic events on brain structure and function are extensive and very specific brain regions have been implicated in trauma and PTSD [107,108,109,110,111]. Significant research has been dedicated towards investigating a variety of psychological treatments to address specific types of such neural dysregulation [3,112,113,114,115,116,117].

In the treatment of trauma, by prompting a neutral/pleasant OR, Calatonia shifts the patient’s experience away from defensive states, leading to the re-setting of vigilant states and attentional processes and facilitating the reinstatement of neocortical–limbic interactions.

By enhancing the perception of a stimulus, Calatonia also activates motivational (or appetitive) systems that support survival, adaptation and tending to one’s needs and, consequently, attentional processes based on ‘interest’, ‘curiosity’ and ‘well-being’. This seemingly simple process conceals a complex reorganization of the individual on physical, emotional and cognitive levels—a process of great psychotherapeutic utility [28,29,30,32,33,34,112].

In terms of its general application in psychotherapy, the OR has been hypothesized as one of the key drivers for successful clinical outcomes following eye movement desensitization and reprocessing (EMDR) [117]. This technique aims to gradually expose the patient to the stimuli underlying PTSD and other trauma disorders, similar to other forms of cognitive-behavioral therapy. Hypothetically, EMDR pairs the recall of a traumatic event with a supposedly emotionally-neutral motor stimulus—eye movements. EMDR appears to show similar improvements in post-therapy outcomes to other cognitive-behavioral therapies particularly for the treatment of PTSD, however the functional mechanisms underlying its action remain unclear [117,118,119,120].

To many patients who suffer from PTSD, the idea of re-experiencing the trauma as proposed in EMDR is unbearable. Unlike EMDR, Calatonia does not target a specific event or memory. Consequently, there is no resetting of new homeostatic values based on previous traumatic experiences, thus amplifying the effects of Calatonia beyond specific trauma memories. Instead, there is a decrease in startle and defensive reflexes and a reinforcing of the “benign present”, allowing the individual to be spontaneously released into a ‘stream of consciousness’ state corresponding to the emergence of the default mode network (DMN) of broad frontoparietal activation in the brain [17]. In this context, high-priority psychological issues may emerge spontaneously and rescript themselves in light of this new experience, producing the myriad of idiosyncratic reports that demonstrate the nonlinearity of psychological processes, followed by a sense of well-being [30].

## 10. Habituation: Does Repeated Calatonia Cease to Generate an OR?

What happens when Calatonia is repeated on a weekly basis? Does it lose its novel impact and stop triggering an OR response?

First, a description of the technique and the consent given by the patient are always requirements to minimize the possibility of a startle or defensive response by making the technique ‘cognitively safe’ and, evidently, this technique is offered only once a good therapeutic relationship is formed. However, a description of this technique does not prevent an OR from occurring as the OR is a result of the direct tactile stimulus and frequently resistant to top-down modulation.

In time, the sequence of touches becomes predictable and thus provides a sense of safety to the patient. Most importantly, what happens within one’s mind, body and emotions during each Calatonia session may become an element of significance for an OR. The route taken towards eliciting an OR may be the same, but the journey is always different. This approach places emphasis on the significance of the event and its capacity to continue to generate a significant OR response. It is very common for patients who receive regular Calatonia to say, “today was different, I didn’t feel the same way I felt last week”, “today the touch seemed much lighter”, “the left side of my body seemed to be heavier”, or similar such observations. These comparisons can be accounted for by Friston’s free energy principle [121], in which the brain is constantly trying to predict events to minimize errors. In this hypothesis, bottom-up processes are thought to compare previous events (memories) to current perceptual inputs to estimate the error in deviation between the internal model and novel input, thus recognizing minimal differences in deviation. Any changes in the representation or original “neural model” of an event that triggered an OR will retrigger the OR by establishing a comparison to what was previously experienced [122,123].

## 11. A Clinical Vignette

The reorganization of the (appetitive) motivational system prompted by Calatonia can be seen in action in a clinical case presenting a dysfunction of the primary motivational behavior for survival, eating. For three consecutive sessions, a fifteen-year-old female patient suffering from anorexia nervosa reported that she “knew exactly what she was going to eat for dinner”. She proceeded to describe to the therapist the meal that came to her mind during the session of Calatonia.

She spontaneously sent pictures of her meals to the therapist shortly following these sessions. These included pictures of her breakfast on the mornings following therapy, revealing nutritious and complete meals. It can be hypothesized that the neurobiological mechanisms set in motion by ORs bypassed her voluntary resistance to homeostatic self-regulation and allostatic behaviors [17], restoring a biological imperative, via “neural circuits in the mammalian brain that prompt specific somatic and autonomic responses associated with motivated behavior” [9].

As the sessions of Calatonia progressed, the sadness and depression she felt surrounding her self-destructive behavior emerged—and her menstrual periods returned, along with these emotions. In her ninth session, she reported a dream in which she “had been kidnapped by a skinny and weak man, from whom she escaped to a shopping mall together with a beautiful girl of the same age who was also his hostage”.

The attentional and motivational processes set in motion during Calatonia seemed to have redirected her perception of her physical needs, revealing the pain she inflicted on herself. In the dream, the self-destructive dynamics that required her awareness were depicted by the skinny and weak man (anorexic thought patterns) and the beautiful girl (her idealized version of herself). She also began to go out more often instead of staying at home watching videos of other anorexic girls. This process suggests what Schomaker and Meeter [124] describe as an “attentional response to novelty, possibly mediated by the amygdala, an arousal-like response to deviance, which could be mediated by the noradrenergic system and a slower upregulation of exploration, motivation and learning, mediated by the dopaminergic system”, as well as a possible reorganization of thalamic functional connectivity [125,126].

## 12. Conclusions

Calatonia and other ST techniques appear to function through the re-calibration of a subset of attentional processes. These include a reduction in the startle response to anxiety- and fear-inducing stimuli and may help to orient the patient towards novel unknown stimuli in a context of adaptation. The redirection of alerting and defensive responses towards motivational and appetitive states through innocuous, pleasant and unusual touch sequences allows the patient to implicitly process past states of trauma. A necessary prerequisite for this technique to be considered a safe psychotherapeutic approach is for the psychotherapist to have adequate training and observe strict adherence to the established protocol of touch and engagement with the patient.

Many studies discuss whether the novelty or significance of an event is the actual trigger of an OR and the consensus is that both novelty and significance are involved in the generation of an orienting response. However, significance was found to be a key factor in sustaining engagement in an OR [7,127], only a relevant/significant novel stimulus will continue to trigger an OR.

In summary, novelty-driven stimulation can support reward processes, drive exploration and other adaptive cognitive processes and enhance perception and sharpen its responses. Most importantly, an OR has a lasting and strong impact on memory and on the attentional system mediated by the amygdala, resulting from neural plasticity and deep changes to the motivational system [124].

## 13. Final Considerations

Beyond the impact of orienting reflexes, there are several other neurobiological, neuro-cognitive and neuro affective-emotional elements that influence the results and responses to the complex stimuli proposed in Calatonia, as listed in a previous publication [30] and briefly mentioned below.

Among these elements, dyadic regulation proposes a fine-tuned, non-verbal, inter-brain synchronization, whereby inter-brain synchronization between two individuals is defined as a natural occurrence that impacts interpersonal communication [128,129,130,131,132].

The importance of establishing a conscious pace of communication in therapeutic relationships cannot be emphasized enough [133,134,135] as several physiological systems follow a preset tempo or rhythm (heartbeat, respiration, thalamo-cortical oscillations) and “rhythms are a prominent signature of brain activity” [136]. The modulation of cortical oscillation via paced somatosensory stimuli may also facilitate integration of the individual’s basic notion of selfhood. From early infancy to adulthood, selfhood is built through physical contact and proximal interaction with others via skin-to-skin interactions—before one develops the ability to share mental states in distal face-to-face interactions [137,138,139,140].

Other hypotheses about the possible elements involved in the complex mental stimuli and contexts observed in Calatonia include:

The use of (task-free) resting-state functional connectivity to facilitate access to spontaneous and pertinent (to psychotherapy) self-reflective cognitive processes [141,142];

(a) The modulation of global brain connectivity and patterns of synchronization (identified as an aspect of brain self-regulation) through the rhythmic segregation and integration of neural populations acting in concert to code for complex stimuli [143,144,145,146,147,148,149];
(b) The engagement of cross-hemispheric communication via the corpus callosum, which facilitates integrative higher-order neural network processes and is implicated in the ability to verbally identify, interpret and communicate emotions [150,151,152,153,154].
(c) The simultaneous engagement of low threshold (sensitive to light touch) skin receptors from the affective-affiliative system in the mammalian nervous system, primarily composed of C-tactile fibers and/or receptors [155,156,157,158,159], polymodal C-receptors, unmyelinated free nerve endings [160] and the low threshold discriminative-spatial system, associated with Merkel’s cell–neurite complex receptors and Ruffini corpuscle proprioceptors [47,137,161,162,163,164,165].
(d) The combination of attentional systems engaged in processing the location and quality of touch, particularly the midline fronto-parietal system activated by the task-positive network, an associative network with extensive bilateral connections with other areas of the brain [127,144,166];

In conclusion, there is significant support for the importance of integrating the orienting reflex in psychotherapy through both physical and non-physical cues. ORs appear to play a mediating role in the improved behavioral outcomes from Calatonia, by initially restoring psychophysical regulation and well-being—and eventually leading to a more positive sense of self. In patients with a history of trauma or attachment issues, this may mean establishing a context of safety within individual boundaries first, through dyadic regulation, before addressing psychological issues that may lead to more feelings of vulnerability [1,167].
